# Geological events and Pliocene climate fluctuations explain the phylogeographical pattern of the cold water fish *Rhynchocypris oxycephalus* (Cypriniformes: Cyprinidae) in China

**DOI:** 10.1186/s12862-014-0225-9

**Published:** 2014-10-25

**Authors:** Dan Yu, Ming Chen, Qiongying Tang, Xiaojuan Li, Huanzhang Liu

**Affiliations:** The Key Laboratory of Aquatic Biodiversity and Conservation, Institute of Hydrobiology, Chinese Academy of Sciences, Wuhan, 430072 P.R. China

**Keywords:** Phylogeography, Cryptic subspecies, Three steps, Neogene climate fluctuations, Conservation

## Abstract

**Background:**

*Rhynchocypris oxycephalus* is a cold water fish with a wide geographic distribution including the relatively warm temperate regions of southern China. It also occurs in second- and third-step geomorphic areas in China. Previous studies have postulated that high-altitude populations of *R. oxycephalus* in southern China are Quaternary glacial relics. In this study, we used the mitochondrial gene *Cytb* and the nuclear gene *RAG2* to investigate the species phylogeographical patterns and to test two biogeographic hypotheses: (1) that divergence between lineages supports the three-step model and (2) climatic fluctuations during the Quaternary resulted in the present distribution in southern China.

**Results:**

Phylogenetic analysis detected three major matrilines (A, B, and C); with matrilines B and C being further subdivided into two submatrilines. Based on genetic distances and morphological differences, matriline A potentially represents a cryptic subspecies. The geographic division between matrilines B and C coincided with the division of the second and third geomorphic steps in China, suggesting a historical vicariance event. Pliocene climatic fluctuations might have facilitated the southwards dispersal of *R. oxycephalus* in matriline C, with the subsequent warming resulting in its split into submatrilines C1 and C2, leaving submatriline C2 as a relic in southern China.

**Conclusions:**

Our study demonstrates that geological events (three steps orogenesis) and climate fluctuations during the Pliocene were important factors in shaping phylogeographical patterns in *R. oxycephalus*. Notably, no genetic diversity was detected in several populations, all of which possessed unique genotypes. This indicates the uniqueness of local populations and calls for a special conservation plan for the whole species at the population level.

**Electronic supplementary material:**

The online version of this article (doi:10.1186/s12862-014-0225-9) contains supplementary material, which is available to authorized users.

## Background

Both geological events and climatic fluctuations usually influence genetic variation distribution in populations and species. The uplifting of mountain systems and the development of river systems may form vicariance between populations, resulting in genetic diversification and even speciation [1-5]. China has a complex topography; the terrain gradually slopes from west to east forming a flight of three steps [6]. The first step consists of the Tibetan Plateau, with an average elevation of over 4,000 m above sea level. The second step includes the Yun-Gui, Loess, and Inner Mongolia Plateaus, the Tarim, Jungar, and Sichuan Basins, and varies from 1,000–2,000 m in elevation. The third step, dropping to 500–1,000 m in elevation, refers to the vast area in the east of the Great Hinggan Ridge-Taihang-Wushan-Xuefeng Mountains and mainly consists of hills and plains [6].

These steps remain geographically and ecologically dynamic; geomorphic evolution is responsible for the isolation and differentiation of many plant and animal populations [5,7]. For example, the geographic division of two lineages of Chinese gecko (*Gekko swinhonis*) coincides with a boundary consisting of the Qinling and Taihang Mountains, suggesting a historical vicariance pattern [8].

Global cyclic fluctuations from the Pliocene and through the Quaternary have resulted in periodic habitat expansions and contractions of species ranges, shaping the species distributions and their genetic patterns [9-11]. Species ranges typically shifted southwards during cold glacial periods and northwards during warm interglacial ones [12]. Quaternary climatic fluctuations and associated habitat expansions and contractions have been investigated extensively in extant organisms [10]. In contrast, the impacts of more ancient climatic changes, as far back as the Pliocene, have been largely neglected despite remarkable shifts in global climate and continental ecosystems [13-15]. Studies have demonstrated that Quaternary glacial climatic oscillations are too recent to explain many of the deep genetic divergences observed today [15,16].

Pliocene climatic oscillations have also occurred in China [17]. During cold periods, continental China was mainly affected by cold air masses and glaciation developed in high mountains. In warm periods, the continental climate was dominated by warm humid air masses, causing glaciers in high mountains to retreat. Similar to current global warming projections, warm humid periods had the potential to eliminate cold-adapted organisms [18,19]. Individual population ranges likely shifted upwards along altitudinal gradients [20,21]. Nevertheless, our knowledge of the genetic consequences of Pliocene climatic change in China remains sparse.

The Chinese minnow (*Rhynchocypris oxycephalus*) is a small riverine cyprinid widely distributed in East Asia. They live in cold, running, or still (but well oxygenated) waters, generally inhabiting stream headwaters at high altitude [22,23]. In China, this species occurs in second- and third-step geomorphic areas. They also occur in the temperate regions of southern China where the average annual temperature is approximately 18°C [24]. Therefore, this is an excellent bioindicator species for low-temperature water and a good model species for studying freshwater fish biogeographic patterns in China because of their typically low dispersal ability and restriction to small mountaintop habitats. It has been suggested that high-altitude populations of *R. oxycephalus* in southern China are Quaternary glacial relics [25]; however, this claim remains unsubstantiated because no phylogeographic studies have been conducted on this minnow to date.

In the present study, we employed mitochondrial DNA (mtDNA) and nuclear DNA (nDNA) markers to test hypotheses on the drivers of biogeographic processes in *R. oxycephalus*. Large-scale sampling allowed us to test hypotheses on the evolutionary history and population demographics of *R. oxycephalus*, to determine: (1) whether or not three steps orogenesis provided different ecological regions that led to high- and low-altitude populations and (2) if Quaternary climatic fluctuations resulted in their present geographic distribution in southern China. Finally, we also assessed the level of genetic diversity for all populations and proposed suitable conservation strategies.

## Methods

### Ethics statement

The experiments were performed in accordance with the Ethics Committee of the Institute of Hydrobiology, Chinese Academy of Sciences. The policies were enacted according to Chinese Association for Laboratory Animal Sciences, and coordinated with the Institutional Animal Care and Use Committee (IACUC) protocols.

### Sample collection

**Figure 1:**
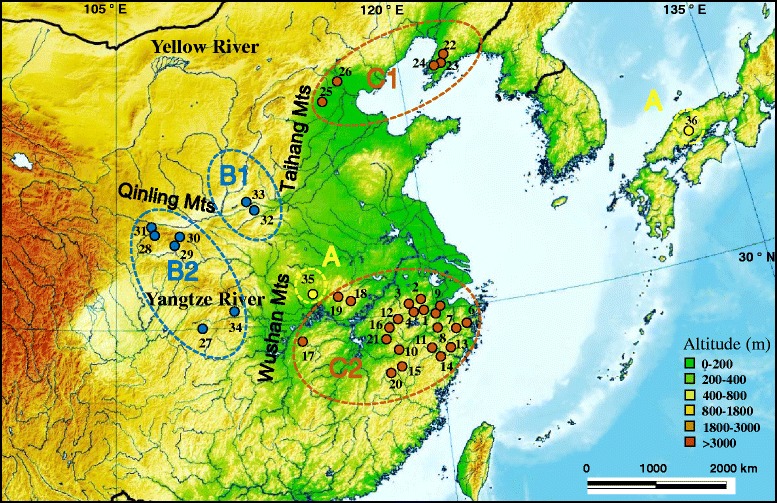
**Map of sampling localities for** ***R*** **.** ***oxycephalus*** **populations.** The map was downloaded from the Wikimedia Commons on May 10, 2014 and slightly modified [26]. The locality numbers are given in Tables 1 and Additional file 1: Table S1. The matrilines are labeled yellow, blue, and brown for matrilines A, B, and C, respectively.

A total of 753 *R. oxycephalus* individuals were collected from 35 localities across most of its distribution in China (Figure 1 and Additional file 1: Table S1). Samples were obtained using a dip net during 2010–2012. All specimens were preserved in 95% ethanol. We aimed to obtain 20–30 samples per site and the median sample size was 23. However, smaller samples were obtained from some localities (Additional file 1: Table S1). We defined samples from different localities as different populations, because they were distributed in different branches of rivers. Four *R. percnurus* and *R. lagowaskii* individuals were used as the outgroup taxa. Detailed information for each specimen used in the present analysis and their localities with GPS records are provided in Additional file 1. All voucher specimens were archived at the Institute of Hydrobiology, Chinese Academy of Sciences.

### DNA extraction, PCR amplification, and sequencing

Total genomic DNA was extracted from muscle tissue following the salt-extraction methods of Tang *et al*. [27]. The mitochondrial cytochrome b gene (*Cytb* 1,140 bp) was amplified for all samples with primers the GluF (5′-AAC CAC CGT TGT ATT CAA CTA CAA-3′) and ThrR (5′-ACC TCC GAT CTT CGG ATT ACA AGA CCG-3′) [28]. Two *Cytb* sequences from Japan were also added to the present study [Genbank: AB236730 and AB198971]. The nuclear Recombinase Activating Gene 2 (*RAG2*) encodes components of the recombinase involved in immunoglobin recombination and T-cell receptor genes and appears as conserved single copies in all vertebrates examined to date [29,30]. Partial *RAG2* sequences (1,127 bp in this study) were obtained from a subset of samples consisting of 127 individuals with the primers RAG2-108f (5′-CCV ARA CGC TCA TGT CCA AC-3′) and RAG2-1324r (5′-TGG ARC AGW AGA TCA TKG C-3′) [31]. Amplifications were performed in a 60-μL reaction volume containing 6 μL of 10 × PCR buffer, 30–50 ng genomic DNA, 1 μL of each primer (each 10 μM), 1.5 μL of dNTPs (each 2.5 mM), and 2.0 U *Taq* DNA polymerase. Sterile water was added to reach the final volume. Reactions took place in the following program: an initial denaturation at 94°C for 3 min, followed by 35 cycles of denaturation at 94°C for 30 s, annealing at 52–58°C for 45 s, extension at 72°C for 1 min, and a final extension at 72°C for 8 min. PCR products were sent to commercial sequencing companies (Shanghai Sangon Biotech Inc.) for purifying and then sequenced in both directions with BigDye™ Terminator Cycle Sequencing Kit on an ABI PRISM® 3730 DNA Analyzer (Applied Biosystems, Life Technologies). The same PCR primers were used for sequencing.

### Sequence analysis

A total of 753 *Cytb* and 127 *RAG2* sequences have been newly sequenced in this study. Multiple alignments of sequences were performed using CLUSTAL X [32] and subsequently checked by eye in SeaView [33]. The nucleotide sequence compositions were calculated in PAUP* v. 4.0b10a [34]. Sequence variations were calculated in MEGA 4.0 [35]. Nuclear gene sequences containing more than one ambiguous site were resolved using PHASE version 2.1.1 [36], for which the input files were prepared using SeqPHASE [37]. Recombination tests for detecting the longest non-recombining region for nuclear loci were conducted in IMGC [38]. Identical haplotypes were collapsed in DnaSP 5.10 [39]. Newly determined sequences were submitted to Genbank [accession numbers KM675061-KM675238] (Additional file 1: Table S1).

### Phylogenetic analysis

Phylogenetic relationships among mitochondrial haplotypes were reconstructed using Bayesian inference (BI), maximum likelihood (ML), and maximum parsimony (MP) approaches. Selection of the best-fit nucleotide substitution models was based on codon position using the Akaike Information Criterion in jmodeltest 2.0 [40,41]. For BI and ML analysis, the most appropriate model of nucleotide substitution was (TIM+I+G). BI analysis was carried out in MrBayes v. 3.1.2 [42]. Four simultaneous Markov chains were run for 6 million generations sampling every 100 generations using two independent analyses in parallel runs. The average standard deviation of split frequencies reached a value below 0.01, the potential scale reduction factor (PSRF) value approached 1.0. After the first 6,000 sampled trees were discarded as burn-in, 50% majority-rule consensus trees with posterior probability values for each node were obtained. ML analysis was conducted with the best model of nucleotide substitution (TIM+I+G) in PhyML 3.0 [43]. Nodal support values were estimated from 100 nonparametric bootstrap replicates. MP analysis was implemented in PAUP 4.0b10a [34]. Heuristic searches with tree-bisection-reconnection were executed for 1,000 random addition replicates with all characters treated as unordered and equally weighted. We constructed the BI tree using *RAG2* alleles under the same settings as for *Cytb*, except for model of substitution (GTR+I+R). Furthermore, the Network 4.6.0.0 program [44] was employed to construct median-joining (MJ) networks to visualize relationships among haplotypes, using the MP calculation set to the default parameters.

### Divergence time estimates

For divergence time estimation, the molecular clock was evaluated using a two-cluster test in the LINTREE program [45]. Sequences that showed significantly different substitution rates were excluded from further analysis. To estimate the divergence time and ancestral distribution of the extant matrilines, we used a coalescent time estimation method in BEAST v 1.7.5 [46]. Major matriline divergence times were estimated using a Yule speciation tree prior. Because of the absence of fossil or geological data for calibrations, divergence times were estimated under the strict-clock model. Based on the mitochondrial *Cytb* data, a range of mutation rates (1–2% per my) were adopted, as they have been widely employed for mitochondrial *Cytb* gene analysis in cyprinid fish [47-50]. Two fixed mutation rates of 1% and 2% were conducted and from these an average divergence time was generated. The credibility interval included the lower limit from the 1% clock and the upper limit from the 2% clock. Analyses were performed for 20 million generations while sampling every 1,000^th^ tree, and the first 10% of trees sampled were treated as burn-in. The effective sample size for parameter estimates and convergence was checked with Tracer 1.5 [51]. FigTree v.1.3.1 [52] was used to display summarized and annotated phylogenetic trees with a molecular clock constraint yielded by BEAST.

### Population genetic analysis

Diversity estimations for both pooled and individual population datasets were calculated in DnaSP, including the number of haplotypes (n), haplotype diversity (h), and nucleotide diversity (π). The mean divergence among haplotypes and divergence between matrilines were calculated by an uncorrected p-distances model as implemented in MEGA, standard errors were estimated by bootstrapping using 10,000 replicates. Pairwise population differentiation was estimated by computing pairwise Φst for the *Cytb* data between populations having more than 10 samples in ARLEQUIN 3.5 [53]. To investigate the level of genetic variation between populations, a two-level hierarchical analysis of molecular variance (AMOVA) was performed. The significance was assessed by 1,000 random permutations.

The plausibility of an isolation-by-distance scenario was explicitly tested by Mantel’s tests in ARLEQUIN to analyze the relationships between genetic (mean number of pairwise nucleotide differences, Φst) and geographical (straight line differences) distances between sampling localities.

### Demographic history

Three methods were used with the *Cytb* data to trace the demographic history of matrilines B1, B2, C1, and C2. Matriline A was excluded form demographic analysis because of the small sample size. First, Tajima’s *D* [54] and Fu’s *Fs* [55] were used to check for neutral evolution of the mtDNA in ARLEQUIN. In the neutrality test, we assessed significance by generating null distributions from 10,000 coalescent simulations; significantly negative values indicated population expansion. Second, mismatch distributions [56] were calculated in ARLEQUIN to test for signals of demographic expansion. For a population undergoing exponential growth, its mismatch distribution should fit a smooth unimodal curve [57]. The significance of sum of squared deviations (SSD) and raggedness indices were carried out by bootstrap resampling (10,000 replicates). Third, Extended Bayesian Skyline Plot EBSP; [58] were implemented in the program BEAST to describe demographic history by assessing the time variation of effective population size. This analysis was performed using the GTR+I+R substitution model and no partition into codon positions. The coalescent tree prior was specified as the Extended Bayesian Skyline Plot. The strict-clock model with mutation rates of 1% per million years was used. The analysis was performed for 20 million generations. The remaining settings were in default mode.

### Morphological differentiation analysis

The ratio of caudal peduncle depth to head length (CPD/HL) and caudal peduncle length to depth (CPL/CPD) are key morphometric variables for discrimination between *R. oxycephalus* and *R. lagowaskii* [59,60]. We measured these two variables for 28 populations (Figure 1: localities 3–12, 14–24, and 27–33) of *R. oxycephalus* and the Xinyang (locality 35) population using digital calipers (IP54 produced by NSCING in Nanjing, China; accurate to the nearest 0.01 mm). Variation of these key morphometric characteristics among the three matrilines (A, B, and C) was subsequently assessed by ANOVA. Tukey’s Honest Significant Difference was employed for *post-hoc* pairwise comparisons. The sample sizes for morphological analysis were 30, 170, and 433 for matrilines A, B, and C, respectively.

## Results

### Sequence data

For *Cytb*, we obtained 755 *R. oxycephalus* sequences from 36 populations, including two sequences from GenBank. The sequence alignment provided a data matrix of 1,097 bp, of which 289 bp (26.3%) were parsimony informative. Base frequencies were unequal (A =25.9%, T =30.4%, C = 27.1%, G =16.6%). Strong compositional biases against G existed at the second and especislly at the third position (just 10.1%). A total of 124 haplotypes were identified among the 755 *R. oxycephalus* sequences (Table 1 and Additional file 1: Table S1). The haplotypes exhibited restricted geographical distributions: 96.7% of the haplotypes were unique for a single location and there were no broadly distributed ones.

**Table 1:** **The collection data for** ***Rhynchocypris oxycephalus*** **samples used in the present study**

**Locality**	**County, Province (Abbr)**	**Lineage**	**n/N**	**Haplotypes**	**h**	**π**
1	Linan, Zhejiang (LA)	C2	2/29	1, 4	0.3400 ± 0.0900	0.0006 ± 0.0002
2	Jiumu, Zhejiang (JM)	C2	2/21	2, 5	0.4670 ± 0.0750	0.0009 ± 0.0001
3	Zhangcun, Zhejiang (ZC)	C2	2/23	6, 8, 12	0.5490 ± 0.0600	0.0033 ± 0.0021
4	Anji, Zhejiang (AJ)	C2	3/10	7, 8, 11	0.5110 ± 0.1640	0.0008 ± 0.0004
5	Lingyin, Zhejiang (LY)	C2	3/23	3, 9, 10	0.1700 ± 0.1020	0.0002 ± 0.0002
6	Fenghua, Zhejiang (FH)	C2	4/16	13–15, 18	0.6420 ± 0.0810	0.0008 ± 0.0002
7	Shengzhou, Zhejiang (SZ)	C2	4/26	16, 17, 19, 21	0.2220 ± 0.1060	0.0004 ± 0.0002
8	Tonglu, Zhejiang (TL)	C2	7/20	20, 22–27	0.7320 ± 0.0920	0.0059 ± 0.0019
9	Daqinggu, Zhejiang (DQG)	C2	1/28	27	—	—
10	Kaihua, Zhejiang (KH)	C2	5/23	28–30, 35, 36	0.7390 ± 0.0490	0.0018 ± 0.0002
11	Wuyi, Zhejiang (WY)	C2	4/26	31, 32, 38, 41	0.2220 ± 0.1060	0.0006 ± 0.0003
12	Shexian, Anhui (SX)	C2	2/22	53, 54	0.0910 ± 0.0810	0.0004 ± 0.0004
13	Panan, Zhejiang (PA)	C2	4/26	33, 34, 37, 39	0.4430 ± 0.1040	0.0004 ± 0.0001
14	Jinyun, Zhejiang (JY)	C2	1/26	40	—	—
15	Shangrao, Jiangxi (SR)	C2	1/27	56	—	—
16	Huangshan, Anhui (HS)	C2	1/14	52	—	—
17	Chibi, Hubei (CB)	C2	8/28	42–49	0.7010 ± 0.0750	0.0017 ± 0.0004
18	Yuexi, Anhui (YX)	C2	1/30	51	—	—
19	Jinzhai, Anhui (JZ)	C2	1/26	55	—	—
20	Wuyishan, Fujian (WYS)	C2	1/21	50	—	—
21	Wuyuan, Jiangxi (WU)	C1	1/12	57	—	—
22	Gaizhou, Liaoning (GZ)	C1	27/30	68–79, 102–114, 122, 123	0.9930 ± 0.0110	0.0068 ± 0.0004
23	Yangyun, Liaoning (YY)	C1	2/3	84, 120	0.6670 ± 0.3140	0.0006 ± 0.0003
24	Shenzijie, Liaoning (SZJ)	C1	3/3	102, 115, 116	1.0000 ± 0.2720	0.0079 ± 0.0025
25	Laishui, Hebei (LS)	C1	1/1	119	—	—
26	Huairou, Beijing (HR)	C1	11/28	80–83,85–88, 117, 118, 121	0.8780 ± 0.0400	0.0020 ± 0.0003
27	Lichuan, Hubei (LC)	B2	1/30	65	—	—
28	Jialingjiang, Shanxi (JLJ)	B2	2/32	59, 62	0.0710 ± 0.0650	0.0008 ± 0.0007
29	Fuping, Shanxi (FP)	B2	2/22	58, 61	0.0910 ± 0.0810	0.0002 ± 0.0002
30	Zhouzhi, Shanxi (ZZ)	B1	2/3	63, 64	0.6670 ± 0.3140	0.0006 ± 0.0003
31	Baoji, Shanxi (BJ)	B2	1/23	60	—	—
32	Mianchi, Henan (MC)	B1/B2	6/27	90–92, 100, 101, 124	0.7070 ± 0.0560	0.0147 ± 0.0051
33	Yuanqu, Shanxi (YQ)	B1/B2	6/29	91, 95–99	0.7340 ± 0.0540	0.0047 ± 0.0032
34	Zigui, Hubei (ZG)	B2	2/16	93, 94	0.4580 ± 0.0950	0.0033 ± 0.0007
35	Xinyang, Henan (XY)	A	1/29	89	—	—
36	Japan, Japan (JP)	A	2/2	66, 67	1.0000 ± 0.5000	0.0173 ± 0.0087

n, the number of *Cytb* haplotypes; N, the number of individuals; h, haplotype diversity; π, nucleotide diversity.

For *RAG2*, we sequenced 127 individuals from a subset of samples selected on the basis of the *Cytb* matrilines (Additional file 1: Table S1). Fourteen individuals were heterozygous. The 1,127-bp fragment included 89 polymorphic sites. All fragments consisted of 62 alleles.

### Matrilineal genealogy and nDNA allele tree

**Figure 2:**
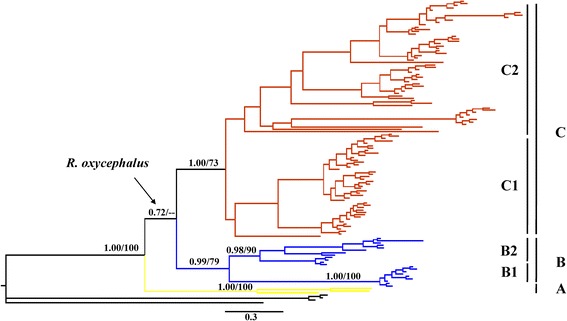
***R*** **.** ***oxycephalus*** **maternal genealogy from Bayesian inference analysis.** Numbers above branches are Bayesian posterior probabilities and bootstrap values from the maximum likelihood analysis, which are only shown for major and minor matrilines. The three major matrilines are identified by different colors (yellow, matriline A; blue, matriline B; and brown, matriline C).

Based on the mtDNA data, three methods (BI, ML, and MP) yielded congruent topologies and they consistently supported three matrilines (the BI tree is shown in Figure 2). Matriline A, at the base of the tree, corresponded to two individuals from Japan and samples from Xinyang (locality 35) belonging to the Huaihe River. It exhibited high divergence from the other matrilines (divergence = 6.9–7.7%). Matriline B, sister to matriline C, contained two submatrilines, one on the east of the Middle Yellow River (B1) and the other on the west of the same river and the Upper Yangtze River tributaries (B2). Matriline C was also subdivided into two submatrilines but with poor node support. Submatriline C1 was composed of individuals from Liaohe River and Haihe River streams in northern China, while submatriline C2 was widely distributed, mainly in the Middle and Lower Yangtze River streams and in Zhejiang Province, southern China.

**Figure 3:**
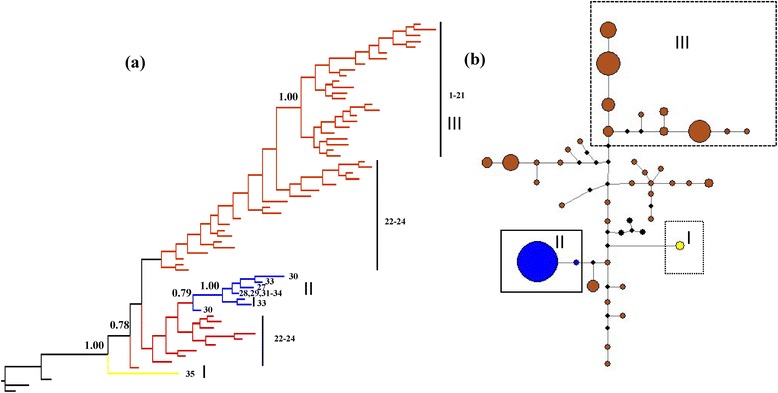
**Allele tree and MJ network for** ***R*** **.** ***oxycephalus*** **based on the nuclear gene** ***RAG2*** **.** The colors correspond to matrilines in Figure 2 (yellow, matriline A; blue, matriline B; and brown, matriline C). The small black rhombus represents each mutational step. **(a)** A Bayesian tree detected three allelic groups I, II, and ІІІ. Numbers above the nodes are Bayesian posterior probabilities. **(b)** MJ network. Three clusters that correspond to the allelic groups in **(a)** are also identified.

For the *RAG2* data, both the BI tree and MJ network showed strong historical patterning: three well-supported groups of alleles, І, ІІ, and ІІІ, were identified (Figure 3a and b). Group I contained all Xinyang individuals (Figure 1: locality 35) corresponding to matriline A (Figure 2) from the Huaihe River. Group II comprised matriline B individuals (Figure 2) (Figure 1: localities 27–34) from the Middle Yellow and Upper Yangtze Rivers. Interestingly, Group II clustered with some individuals from matriline C1. Group ІІІ consisted of samples from matriline C2 (Figure 2) (Figure 1: localities 1–21) from the Middle and Lower Yangtze River basin and Zhejiang Province. The relationships of the individuals in matriline C1 were unresolved.

### Divergence time estimate

**Figure 4:**
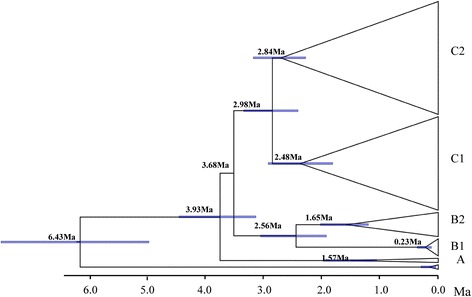
**Divergence time tree for** ***R*** **.** ***oxycephalus.*** Branch lengths are proportional to divergence times. Bars on the nodes are 95% confidence intervals. Matrilines A, B1, B2, and C1, C2 correspond to those in Figure 2.

The two-cluster test showed that three sequences (haplotypes 22–24, locality 8) had significantly different substitution rates compared with the other sequences. Therefore, these three sequences were excluded from further analysis. A strict molecular clock was conducted for the remaining samples (Figure 4). Time since the most recent common ancestor of the entire ingroup was estimated to be 6.43 mya (4.30–8.61 mya). Matriline A diverged 3.93 mya (2.62–5.25 mya) and matrilines B and C diverged 3.68 mya (2.45–4.92 mya). Submatrilines C1 and C2 diverged 2.98 mya (1.98–3.99 mya).

### Molecular diversity and genetic structure

The overall *Cytb* haplotype (h) and nucleotide (π) diversities in all samples were 0.9770 ± 0.0010 and 0.0523 ± 0.0006, respectively. The number of haplotypes (n), h, and π varied among populations (Table 1). The Gaizhou population (Figure 1: locality 22), which exhibited the highest h value, contained haplotypes from submatriline C1. The Mianchi population (Figure 1: locality 32) had the highest π value, which corresponded to the co-occurrence of haplotypes from submatrilines B1 and B2. In contrast, 12 populations (Daqinggu, Jinyun, Shangrao, Huangshan, Yuexi, Jinzhai, Wuyishan, Wuyuan, Laishui, Lichuan, Baoji, and Xinyang, corresponding to localities 9, 14–16, 18–21, 25, 27, 31, and 35) each contained only a single haplotype. The two-level AMOVA suggested that *R. oxycephalus* was highly geographically structured: 96.75% of the genetic variation was attributed to differentiation among populations and this was highly significant (*P* <0.001). All pairwise Φst values were statistically significant, varying from 0.099 to 1.000 (*P* <0.001 to *P* = 0.027). Genetic divergence varied from 6.5 to 7.4% among matrilines A, B, and C, and from 5.5 to 5.6% between submatrilines B1-B2 and C1-C2 (Table 2).

**Table 2:** **Genetic distance for** ***Cytb*** **between major and minor matrilines based on the uncorrected p-distances model**

	**A**	**B**	**C**	**C1**
A				
B	0.070			
C	0.074	0.065		
C1	0.069	0.060	—	
C2	0.077	0.069	—	0.055

A significant positive correlation was found between geographical distance and genetic divergence among populations (for *Cytb*: *r* = 0.30, *P* <0.01; for *RAG2*: *r* = 0.25, *P* <0.01). However, some populations (e.g., Wuyuan and Huangshan, Wuyishan and Shangrao, Baoji, and Jialingjiang, corresponding to localities 21 and 16, 20 and 15, 28 and 31, respectively) were genetically distinct despite their geographical proximity. These populations are distributed on opposite sides of the same mountains.

### Demographic history

For submatriline B1, Tajima’s *D* and Fu’s *Fs* were negative but not significant (Table 3). However, values of SSD and raggedness index did not reject the hypothesis of sudden expansion and the B1 mismatch distribution was unimodal (Additional file 2: Figure S1a). Extended Bayesian Skyline Plots (EBSP) suggested that the effective population size was slowly increasing (Additional file 3: Figure S2a). For submatrilines B2, C1, and C2, population expansions were not supported in neutrality tests (Table 3) and the mismatch analyses significantly rejected the expansion hypothesis. However, the EBSP suggested a decline followed by a steep increase in effective population size for submatrilines C1 and C2 (Additional file 3: Figure S2b and d) and a steep decline for submatriline B2 (Additional file 3: Figure S2c). Therefore, in all matrilines there was ambiguity in the demographic scenarios suggested by all three methods.

**Table 3:** **Statistics of neutrality tests and mismatch distribution analysis for matrilines**

	**Neutrality tests**		**Mismatch distribution**	
	**Tajima’s D**	**Fu’s** ***Fs***	**SSD**	**Raggedness**
**Matriline**	**(** ***P*** **-value)**	**(** ***P*** **-value)**	**(** ***P*** **-value)**	**Index (** ***P*** **-value)**
Matriline B1	−0.3057 (0.4150)	−0.7241 (0.3730)	0.0067 (0.7000)	0.0222 (0.9500)
Matriline B2	1.3143 (0.9370)	26.2444 (1.0000)	0.0438 (0.0300)	0.0345 (0.0100)
Matriline C1	0.9444 (0.8670)	−3.7961 (0.1970)	0.0233 (0.0500)	0.0069 (0.2800)
Matriline C2	0.9266 (0.8600)	26.7796 (0.9800)	0.0093 (0.0400)	0.0067 (0.0000)

SSD, sum of squares deviations.

### Morphological differentiation

The ANOVA analysis showed that two key taxonomic characteristics varied significantly among the three matrilines (CPD/HL: *df* = 2, 630, *P* = 0.000; CPL/CPD: *df* = 2, 630, *P* = 0.001). For CPD/HL, there was no significant difference between matrilines A and B (Tukey’s *post*-*hoc* tests, *P* = 0.607). However, significant pair-wise distinctions were detected between matrilines A and C and between matrilines B and C (Tukey’s *post*-*hoc* tests, *P* = 0.017 and *P* = 0.001, respectively). For CPL/CPD, matriline A values differed significantly from those of B and C (Tukey’s *post*-*hoc* tests, *P* = 0.027 and *P* = 0.001, respectively), but there was no significant difference between matrilines B and C (Tukey’s *post*-*hoc* tests, *P* = 0.202).

## Discussion

### Deep matriline divergence and implication of cryptic subspecies

Phylogenetic analysis based on *Cytb* revealed three matrilines (Figure 2): these are highly consistent with the geographic structure of the *R. oxycephalus* range. Matriline A was first to split from the other two and exhibits high divergence from them. The Taihang-Wushan Mountains seem to act as dividing lines between matrilines B and C. Further divergence occurred between populations in matriline B (B1 and B2) and C (C1 and C2). The nDNA phylogenetic and network analysis revealed a deep genetic divergence analogous to mtDNA (Figure 3).

There is a deep genetic divergence between matriline A and the other matrilines for *Cytb* (6.9–7.7%, Table 2). Compared with other cyprinid fishes, this amount of divergence has been attributed to subspecies-level differences [61]. The *RAG2* tree results were consistent with those from the *Cytb* tree, matriline A is basal in the BI tree and the MJ network, indicating that matriline A is most distant from the others (Figure 3). We also sequenced some *Cytb* and *RAG2* sequences from other closed *Rhynchocypris* species (e.g., *R. percnurus*, *R. lagowaskii*, and *R. czekanowskii*). The results revealed that matriline A is a distinct monophyletic group and forms a sister relationship with *R. oxycephalus*. Furthermore, samples from Xinyang (locality 35), representing matriline A in our samples, are morphologically distinct from the other populations in taxonomically relevant ratios. Therefore, we suggest that matriline A represents a cryptic subspecies, which may be geographically restricted to Japan and parts of China including the Huaihe River. Although Japan is currently separated from eastern China, studies have proposed that these regions most likely had the same freshwater ichthyofauna during the Miocene and Pliocene [62].

The *Cytb* sequences analysis carried out in our study revealed three distinct evolutionary lineages in *R. oxycephalus*. Do these major lineages correspond to two or three distinct species? Compared with the general definitions of five different groups of candidate species [63], all uncorrected *p*-distances between the *R. oxycephalus* lineages reached a value associated with subspecies divergences (including matrilines B and C). Additionally, morphological differences (CPD/HL) were also observed between the major lineages (matrilines A and C, matrilines B and C). However, the relationship between matrilines B and C is not completely supported by the *RAG2* gene data, but matriline A is indeed distinct from the others (as discussed in the previous paragraph). Therefore, the hypothesis of matrilines B and C being distinct subspecies or even species requires further research.

### Geologically driven divergence between matrilines B and C

The deep divergence observed between matrilines B and C likely reflects long-term isolation and ecological differences between high- and low-elevation regions, which is probably associated with China’s complex geological history. As indicated in Figure 1, matriline B populations are mainly distributed in high-altitude areas; while matriline C occurs in the lowland regions. The Taihang-Wushan Mountains, part of the demarcation line for the second- and third-step geomorphic areas in China [15], separate matrilines B and C. Therefore, *R. oxycephalus* divergence is likely associated with the orogenesis of China’s three steps. Geological evidence indicates that recent large-scale intense uplift of the Qinghai-Tibetan Plateau took place during the Pliocene 3.6 mya [64], contributing to the formation of three steps land features [65]. During this period, new habitats and different ecological regions may have been created leading to upper and lower populations. These ecological differences may have resulted in high levels of divergence between matrilines B and C, fostering the accumulation of autapomorphic mutations. The mean estimate of divergence between matrilines B and C was 3.68 mya (2.45–4.92 mya in the Pliocene), suggesting that the speciation event occurred at approximately the same time as the geological event. Similar effects have been reported for several species of plants [3] and animals [66,67]. Based on the *Cytb* gene sequences, He and Chen [68] suggested that the highly specialized schizothoracine fishes may have originated in the early Pliocene and the major cladogenetic events in this species are closely correlated with marked environmental changes caused by the violent upheaval of the plateau. The geographic division between two Chinese gecko (*Gekko swinhonis*) lineages also coincides with the Qinling and Taihang Mountains boundary, suggesting a historical vicariance pattern. The orogeny of the Qinling Mountains may have launched the independent lineage divergence [8]. Thus, barriers to gene flow produced by the complex geological history appear to be responsible for driving the high level of species diversity in the mountains of China.

### Pliocene climate fluctuation driven split between submatrilines C1 and C2

There is a 5.5% mtDNA difference between submatrilines C1 and C2 (Table 2). This high level of divergence likely reflects long-term isolation between the two submatrilines. Our molecular clock analysis estimated that submatriline C1 split from C2 approximately 2.98 mya (1.98–3.99 mya), corresponding to the Pliocene warming (2.6–5.0 mya) [69-72]. This warming period was preceded by an extensive cooling (5.5–5.2 mya) [14,71]. During the colder period, matriline C may have extended its range southwards to southern China, resulting in a widespread distribution of the species. The subsequent warming then resulted in the split of matriline C into submatrilines C1 and C2; submatriline C1 is now distributed in cold northern China, while C2 remains in southern China. Similar effects have been reported in speckled dace (*R. osculus*) in western North America [73]. In Asian salamanders (*Pachytriton*), all major intraspecific lineages originated before the Quaternary period and pre-Quaternary climate change played an important role in the formation of regional biodiversity [15].

After the split of submatrilines C1 and C2, intraspecific divergence may have been intensified through cyclical climatic fluctuations in the Pleistocene, which also facilitated the relatively wide distribution of *R. oxycephalus* in southern China. Similar processes have been described in terrestrial montane organisms. Thus, our conclusion is inconsistent with the hypothesis that *R. oxycephalus* populations in southern China are Quaternary glacial relics in the high-altitude mountains of this region [25].

### Species-level genetic diversity and structure

Chinese *R. oxycephalus* populations are highly genetically diverse. For the *Cytb* gene in all samples, there were 289 (26.3%) parsimony informative sites and the most variable sites occurred in different haplotypes. AMOVA analysis revealed that 96.75% of the genetic variation was attributed to differentiation among populations. In contrast, genetic diversity within populations was very low and 12 populations possessed a single haplotype despite typically analyzing over 20 fish per population. These populations may have had extremely small founding populations. A similar phenomenon has been reported in *Quasipaa boulengeri*, a montane stream-dwelling frog, where 85% of the haplotypes only occurred in a single location and 16 populations out of 45 had zero haplotype and nucleotide diversity [74]. High levels of genetic diversity among populations and low levels within populations may commonly occur in montane streams [75]. In these species, local extinctions may considerably impoverish species genetic diversity via the loss of unique haplotypes.

The genetic structure of a species is greatly influenced by the palaeogeographical history of the region that comprises its geographical range. In the present study, mtDNA analysis suggested that *R. oxycephalus* was geographically restricted to local montane areas. Approximately 97% of the haplotypes are unique for their respective populations. The majority of genetic variance was seen at the population level and was correlated with geographic distance; more distant sites within the hydrological network were more genetically differentiated. In addition to complex topological changes, this pattern may have resulted from the isolation of populations because of specific habitat requirements. *Rhynchocypris oxycephalus* mainly occurs in the clear cold permanent headwaters of montane streams. This specific ecological requirement results in small population sizes and restricts gene flow among populations (each population possesses unique haplotypes). Therefore, these populations most likely survive in isolated habitats and differentiate through genetic drift and selection.

### Implications for conservation

The Chinese minnow currently has a wide geographical distribution in East Asia and to date is ranked as ‘not threatened’. However, individual populations are small because they are restricted to stream headwaters and may be sensitive to climate change and anthropogenic threats. Using ecological niche modeling, Yu *et al*. [24] predicted that climate change poses a severe threat to *R. oxycephalus*. The geographical distribution of this species is expected to contract with progressing climate change and become severely limited, particularly in south-eastern China. Anthropogenic threats, such as deforestation, illegal fishing, and habitat destruction (e.g., road construction and hydro-electric power station) may represent major threats to this species. These factors place *R. oxycephalus* at risk for extinction.

Genetic variability is widely recognized as an important component of natural biodiversity. The maintenance of high genetic diversity could prevent the loss of a species evolutionary potential [76]. The distinct genetic differentiation in *R. oxycephalus* has important implications for the conservation of this species. However, many *R. oxycephalus* populations inhabit areas that are not protected. Our study may serve as a guide for monitoring and developing a conservation strategy for this species. Within this framework, our analyses suggest that the three matrilineal ranges (B, C1, and C2) be considered individual Management Units. All of these require protection because of their genetic uniqueness, which includes exclusive mtDNA haplotypes and private nuclear alleles.

Additionally, the southern populations including those from the Yangtze River, Zhejiang Province, and the Min River demand special attention. This species is geographically restricted to local streams and many of these populations possess negligible genetic diversity. Overall genetic diversity in *R. oxycephalus* will be reduced if these populations become extinct. Climate change projections indicate severe threats to *R. oxycephalus* and to these southern populations in particular [24]. Consequently, we recommend that conservation efforts should pay particular attention to areas with unique genetic information. Long-term monitoring programs should also be established, irrespective of whether local population genetic diversity is high or low.

## Conclusions

This study assembles a large dataset to infer *R. oxycephalus* phylogeographic patterns in China. The results support the hypothesis that geological events (three steps orogenesis) have driven divergence between matrilines B and C. Based on the molecular clock, it is unlikely that climate fluctuations during the Quaternary facilitated the southwards dispersal of *R. oxycephalus* in matriline C and established relics in south China as postulated by Zhang and Chen [25]. Pliocene cooling and warming might have resulted in the split of submatrilines C1 and C2. Notably, no genetic diversity was detected in several populations and all of these populations possessed unique genotypes. This indicates the uniqueness of local populations and calls for a special conservation concern for this species at the population level. These conclusions are based on *Cytb* and *RAG2* gene data; analysis with additional markers would greatly enhance our understanding of this species.

## Additional files

Additional file 1: Table S1. Summary of sample localities for *Rhynchocypris oxycephalus* and outgroups. The locality numbers correspond to Figure 1. Site, coordinates (longitude/latitude), sample size, voucher ID number, *Cytb* haplotype and identified lineages, nuclear clade, and GenBank accession number for *Cytb* and *RAG2* are presented. Additional file 2: Figure S1. Mismatch distribution for each *R. oxycephalus* matriline. The abscissa indicates the number of pairwise differences between compared sequences. The ordinate is the frequency for each value. Histograms are the observed frequencies of pairwise divergences among sequences and the line refers to the expectation under the model of population expansion. (a–d) Mismatch distributions for the matrilines B1, B2, C1, and C2, respectively. Additional file 3: Figure S2. Bayesian skyline plot estimated by BEAST for each *R. oxycephalus* matriline. X-axis, time in millions of years; y-axis, effective population size (units = Neτ, the product of effective population size and generation length in millions of years). The mean estimate and both 95% HPD limits are indicated. (a–d) Bayesian skyline plots for matrilines B1, B2, C1, and C2, respectively.
