# Supplementary material for: Geological events and Pliocene climate fluctuations explain the phylogeographical pattern of the cold water fish Rhynchocypris oxycephalus (Cypriniformes: Cyprinidae) in China
Source: BMC Evol Biol. 2014 Oct 25;14:225. doi: 10.1186/s12862-014-0225-9 (PMC4219125; doi:10.1186/s12862-014-0225-9)
Supplement: Additional file 1: Table S1. — Summary of sample localities for Rhynchocypris oxycephalus and outgroups. The locality numbers correspond to Figure 1. Site, coordinates (longitude/latitude), sample size, voucher ID number, Cytb haplotype and identified lineages, nuclear clade, and GenBank accession number for Cytb and RAG2 are presented. [file 12862_2014_225_MOESM1_ESM.pdf]

Table S1 Summary of sampling localities for *Rhynchocypris oxycephalus* and outgroups. The locality numbers correspond to Figure 1. Site, coordinates (longitude/latitude), sample size, voucher ID number, *Cytb* haplotype and identified lineages, nuclear clade, and GenBank accession number for *Cytb* and *RAG2* are presented.

**Species**	**Site (number)**	**Longitude**	**Latitude**	**Sample size**	**Voucher No.**	***Cytb* Hap**	**MtDNA Lineage**	***RAG2* clade**	**GenBank Accession No.**	
									Cytb	***RAG2***
Ingroup										
R. oxycephalus	1	119.67	30.37	29	20101449	hap 1	C2		KM675120	
					20101450	hap 1	C2		KM675120	
					20101451	hap 1	C2		KM675120	
					20101452	hap 4	C2		KM675123	
					20101453	hap 1	C2		KM675120	
					20101454	hap 1	C2		KM675120	
					20101455	hap 1	C2		KM675120	
					20101456	hap 4	C2		KM675123	
					20101457	hap 1	C2	III	KM675120	KM675074
					20101458	hap 4	C2	III	KM675123	KM675074
					20101459	hap 1	C2		KM675120	
					20101460	hap 1	C2		KM675120	
					20101461	hap 4	C2		KM675123	
					20101463	hap 1	C2		KM675120	
					20101464	hap 1	C2		KM675120	
					20101465	hap 1	C2		KM675120	
					20101466	hap 1	C2		KM675120	
					20101467	hap 1	C2		KM675120	
					20101468	hap 1	C2		KM675120	
					20101469	hap 4	C2		KM675123	

				20101470	hap 1	C2		KM675120	
				20101471	hap 1	C2		KM675120	
				20101472	hap 1	C2		KM675120	
				20101473	hap 1	C2		KM675120	
				20101474	hap 1	C2		KM675120	
				20101475	hap 1	C2		KM675120	
				20101476	hap 4	C2		KM675123	
				20101477	hap 1	C2		KM675120	
				20101478	hap 1	C2		KM675120	
2	119.65	30.42	21	20101336	hap 5	C2		KM675124	
				20101337	hap 5	C2		KM675124	
				20101338	hap 5	C2		KM675124	
				20101339	hap 5	C2	III	KM675124	KM675061
				20101340	hap 5	C2		KM675124	
				20101341	hap 5	C2		KM675124	
				20101342	hap 5	C2		KM675124	
				20101343	hap 5	C2		KM675124	
				20101345	hap 5	C2		KM675124	
				20101346	hap 5	C2		KM675124	
				20101347	hap 5	C2		KM675124	
				20101348	hap 5	C2		KM675124	
				20101349	hap 5	C2		KM675124	
				20101350	hap 5	C2		KM675124	
				20101351	hap 2	C2		KM675121	
				20101352	hap 2	C2	III	KM675121	KM675061
				20101353	hap 2	C2		KM675121	

				20101354	hap 2	C2		KM675121	
				20101355	hap 2	C2		KM675121	
				20101356	hap 2	C2		KM675121	
				20101357	hap 2	C2		KM675121	
3	119.41	30.42	23	20101203	hap 6	C2	III	KM675125	KM675110
				20101204	hap 8	C2		KM675127	
				20101205	hap 8	C2		KM675127	
				20101206	hap 8	C2		KM675127	
				20101207	hap 8	C2		KM675127	
				20101208	hap 8	C2		KM675127	
				20101209	hap 6	C2		KM675125	
				20101210	hap 8	C2		KM675127	
				20101211	hap 8	C2	III	KM675127	KM675111
				20101212	hap 8	C2		KM675127	
				20101213	hap 8	C2		KM675127	
				20101214	hap 12	C2		KM675131	
				20101215	hap 6	C2		KM675125	
				20101216	hap 6	C2		KM675125	
				20101217	hap 6	C2		KM675125	
				20101218	hap 6	C2		KM675125	
				20101219	hap 6	C2		KM675125	
				20101220	hap 6	C2		KM675125	
				20101221	hap 6	C2		KM675125	
				20101222	hap 6	C2		KM675125	
				20101223	hap 6	C2		KM675125	
				20101224	hap 6	C2		KM675125	

				20101225	hap 6	C2		KM675125	
4	119.42	30.41	10	20101159	hap 7	C2		KM675126	
				20101161	hap 8	C2		KM675127	
				20101163	hap 8	C2		KM675127	
				20101164	hap 11	C2	III	KM675127	KM675061
				20101166	hap 8	C2		KM675127	
				20101169	hap 8	C2		KM675127	
				20101170	hap 8	C2		KM675127	
				20101171	hap 7	C2	III	KM675126	KM675061
				20101175	hap 8	C2		KM675127	
				20101176	hap 8	C2		KM675127	
5	120.1	30.25	23	1004002	hap 3	C2		KM675122	
				1004003	hap 3	C2		KM675122	
				1004004	hap 9	C2		KM675128	
				1004005	hap 3	C2		KM675122	
				1004007	hap 3	C2		KM675122	
				1004008	hap 10	C2	III	KM675129	KM675067
				1004010	hap 3	C2		KM675122	
				1004012	hap 3	C2		KM675122	
				1004014	hap 3	C2	III	KM675122	KM675067
				1004015	hap 3	C2		KM675122	
				1004016	hap 3	C2		KM675122	
				1004018	hap 3	C2		KM675122	
				1004019	hap 3	C2		KM675122	
				1004020	hap 3	C2		KM675122	
				1004021	hap 3	C2		KM675122	

				1004022	hap 3	C2		KM675122	
				1004024	hap 3	C2		KM675122	
				1004025	hap 3	C2		KM675122	
				1004026	hap 3	C2		KM675122	
				1004027	hap 3	C2		KM675122	
				1004028	hap 3	C2		KM675122	
				1004029	hap 3	C2		KM675122	
				1004031	hap 3	C2		KM675122	
6	121.1	29.71	16	20101253	hap 13	C2		KM675132	
				20101254	hap 15	C2		KM675134	
				20101256	hap 14	C2	III	KM675133	KM675085
				20101257	hap 13	C2		KM675132	
				20101259	hap 13	C2	III	KM675132	KM675071
				20101260	hap 14	C2		KM675133	
				20101261	hap 13	C2		KM675132	
				20101262	hap 14	C2		KM675133	
				20101263	hap 14	C2		KM675133	
				20101264	hap 13	C2		KM675132	
				20101265	hap 13	C2		KM675132	
				20101266	hap 14	C2		KM675133	
				20101268	hap 13	C2		KM675132	
				20101271	hap 13	C2		KM675132	
				20101272	hap 18	C2		KM675137	
				20101274	hap 14	C2		KM675133	
7	120.56	29.63	26	20101131	hap 17	C2		KM675136	
				20101132	hap 17	C2		KM675136	

				20101133	hap 17	C2		KM675136	
				20101134	hap 17	C2		KM675136	
				20101135	hap 19	C2		KM675138	
				20101136	hap 17	C2		KM675136	
				20101137	hap 17	C2		KM675136	
				20101138	hap 16	C2		KM675135	
				20101139	hap 17	C2		KM675136	
				20101140	hap 17	C2		KM675136	
				20101141	hap 17	C2		KM675136	
				20101142	hap 17	C2		KM675136	
				20101143	hap 21	C2		KM675140	
				20101144	hap 17	C2		KM675136	
				20101145	hap 17	C2	III	KM675136	KM675070
				20101146	hap 17	C2		KM675136	
				20101148	hap 17	C2		KM675136	
				20101149	hap 17	C2		KM675136	
				20101150	hap 17	C2		KM675136	
				20101151	hap 17	C2		KM675136	
				20101152	hap 17	C2		KM675136	
				20101153	hap 17	C2		KM675136	
				20101154	hap 17	C2		KM675136	
				20101155	hap 17	C2		KM675136	
				20101156	hap 17	C2		KM675136	
				20101157	hap 17	C2		KM675136	
8	119.71	29.70	20	20101110	hap 24	C2	III	KM675116	KM675067
				20101111	hap 27	C2		KM675143	

				20101112	hap 26	C2		KM675142	
				20101113	hap 22	C2	III	KM675114	KM675073
				20101114	hap 27	C2		KM675143	
				20101115	hap 27	C2	III	KM675143	KM675073
				20101116	hap 26	C2		KM675142	
				20101117	hap 23	C2		KM675115	
				20101118	hap 27	C2		KM675143	
				20101119	hap 26	C2		KM675142	
				20101120	hap 27	C2		KM675143	
				20101121	hap 27	C2		KM675143	
				20101122	hap 27	C2		KM675143	
				20101123	hap 27	C2		KM675143	
				20101124	hap 27	C2		KM675143	
				20101125	hap 25	C2		KM675141	
				20101127	hap 29	C2		KM675145	
				20101128	hap 20	C2		KM675139	
				20101129	hap 20	C2		KM675139	
				20101130	hap 20	C2	III	KM675139	KM675073
9	120.07	30.20	28	1004029	hap 27	C2		KM675143	
				1004030	hap 27	C2		KM675143	
				1004031	hap 27	C2		KM675143	
				1004032	hap 27	C2		KM675143	
				1004033	hap 27	C2		KM675143	
				1004034	hap 27	C2		KM675143	
				1004035	hap 27	C2		KM675143	
				1004036	hap 27	C2		KM675143	

				1004037	hap 27	C2		KM675143	
				1004039	hap 27	C2		KM675143	
				1004040	hap 27	C2		KM675143	
				1004041	hap 27	C2		KM675143	
				1004042	hap 27	C2		KM675143	
				1004043	hap 27	C2		KM675143	
				1004044	hap 27	C2		KM675143	
				1004045	hap 27	C2		KM675143	
				1004046	hap 27	C2		KM675143	
				1004047	hap 27	C2		KM675143	
				1004048	hap 27	C2		KM675143	
				1004049	hap 27	C2		KM675143	
				1004050	hap 27	C2		KM675143	
				1004051	hap 27	C2	III	KM675143	KM675067
				1004052	hap 27	C2		KM675143	
				1004053	hap 27	C2		KM675143	
				1004054	hap 27	C2		KM675143	
				1004055	hap 27	C2		KM675143	
				1004056	hap 27	C2		KM675143	
				1004057	hap 27	C2		KM675143	
				1004058	hap 27	C2		KM675143	
10	118.14	29.11	23	20101358	hap 28	C2	III	KM675144	KM675080
				20101359	hap 29	C2		KM675145	
				20101360	hap 29	C2		KM675145	
				20101361	hap 30	C2		KM675146	
				20101362	hap 30	C2		KM675146	

				20101363	hap 28	C2		KM675144	
				20101364	hap 30	C2		KM675146	
				20101365	hap 29	C2	III	KM675145	KM675066
				20101366	hap 29	C2		KM675145	
				20101367	hap 28	C2		KM675144	
				20101368	hap 28	C2		KM675144	
				20101369	hap 30	C2		KM675146	
				20101370	hap 28	C2		KM675144	
				20101371	hap 30	C2		KM675146	
				20101372	hap 28	C2		KM675144	
				20101619	hap 28	C2		KM675144	
				20101621	hap 36	C2	III	KM675152	KM675086
				20101622	hap 29	C2		KM675145	
				20101624	hap 29	C2		KM675145	
				20101625	hap 28	C2		KM675144	
				20101626	hap 28	C2		KM675144	
				20101627	hap 28	C2		KM675144	
				20101628	hap 35	C2	III	KM675151	KM675080
11	119.83	28.83	26	20101373	hap 32	C2		KM675148	
				20101375	hap 32	C2		KM675148	
				20101376	hap 32	C2		KM675148	
				20101377	hap 32	C2		KM675148	
				20101378	hap 32	C2		KM675148	
				20101379	hap 32	C2		KM675148	
				20101380	hap 32	C2		KM675148	
				20101381	hap 32	C2		KM675148	

					20101382	hap 31	C2	III	KM675147	KM675061
					20101383	hap 32	C2		KM675148	
					20101384	hap 38	C2		KM675154	
					20101385	hap 32	C2		KM675148	
					20101386	hap 41	C2		KM675157	
					20101387	hap 32	C2		KM675148	
					20101388	hap 32	C2		KM675148	
					20101389	hap 32	C2		KM675148	
					20101390	hap 32	C2		KM675148	
					20101391	hap 32	C2		KM675148	
					20101392	hap 32	C2		KM675148	
					20101393	hap 32	C2		KM675148	
					20101394	hap 32	C2		KM675148	
					20101395	hap 32	C2		KM675148	
					20101396	hap 32	C2		KM675148	
					20101397	hap 32	C2		KM675148	
					20101398	hap 32	C2	III	KM675148	KM675061
					20101399	hap 32	C2		KM675148	
12		118.32	30.08	22	20101179	hap 53	C2	III	KM675169	KM675075
					20101180	hap 53	C2		KM675169	
					20101181	hap 53	C2		KM675169	
					20101183	hap 53	C2		KM675169	
					20101184	hap 53	C2		KM675169	
					20101185	hap 53	C2		KM675169	
					20101186	hap 53	C2		KM675169	
					20101187	hap 53	C2		KM675169	

				20101188	hap 53	C2		KM675169	
				20101189	hap 53	C2		KM675169	
				20101190	hap 53	C2		KM675169	
				20101191	hap 53	C2		KM675169	
				20101192	hap 53	C2		KM675169	
				20101193	hap 53	C2		KM675169	
				20101194	hap 53	C2		KM675169	
				20101195	hap 53	C2		KM675169	
				20101196	hap 53	C2		KM675169	
				20101197	hap 54	C2	III	KM675170	KM675075
				20101198	hap 53	C2		KM675169	
				20101199	hap 53	C2		KM675169	
				20101201	hap 53	C2		KM675169	
				20101202	hap 53	C2		KM675169	
13	120.46	29.00	26	20101306	hap 39	C2	III	KM675155	KM675082
				20101307	hap 34	C2		KM675150	
				20101308	hap 34	C2		KM675150	
				20101309	hap 33	C2		KM675149	
				20101310	hap 33	C2		KM675149	
				20101312	hap 33	C2		KM675149	
				20101313	hap 33	C2		KM675149	
				20101314	hap 33	C2		KM675149	
				20101315	hap 33	C2		KM675149	
				20101316	hap 33	C2		KM675149	
				20101317	hap 33	C2		KM675149	
				20101318	hap 33	C2		KM675149	

				20101319	hap 34	C2		KM675150	
				20101321	hap 33	C2		KM675149	
				20101322	hap 33	C2		KM675149	
				20101323	hap 33	C2		KM675149	
				20101324	hap 34	C2		KM675150	
				20101325	hap 33	C2		KM675149	
				20101326	hap 34	C2	III	KM675150	KM675072
				20101327	hap 33	C2		KM675149	
				20101328	hap 33	C2		KM675149	
				20101329	hap 33	C2	III	KM675149	KM675072
				20101331	hap 33	C2		KM675149	
				20101332	hap 33	C2		KM675149	
				20101334	hap 33	C2		KM675149	
				20101335	hap 37	C2	III	KM675153	KM675082
14	120.57	28.51	26	20101226	hap 40	C2		KM675156	
				20101227	hap 40	C2		KM675156	
				20101228	hap 40	C2		KM675156	
				20101229	hap 40	C2	III	KM675156	KM675061
				20101230	hap 40	C2		KM675156	
				20101231	hap 40	C2		KM675156	
				20101232	hap 40	C2		KM675156	
				20101233	hap 40	C2		KM675156	
				20101234	hap 40	C2		KM675156	
				20101235	hap 40	C2		KM675156	
				20101236	hap 40	C2		KM675156	
				20101237	hap 40	C2		KM675156	

				20101238	hap 40	C2		KM675156	
				20101239	hap 40	C2		KM675156	
				20101240	hap 40	C2		KM675156	
				20101241	hap 40	C2		KM675156	
				20101242	hap 40	C2		KM675156	
				20101243	hap 40	C2		KM675156	
				20101244	hap 40	C2		KM675156	
				20101245	hap 40	C2		KM675156	
				20101246	hap 40	C2		KM675156	
				20101247	hap 40	C2		KM675156	
				20101248	hap 40	C2		KM675156	
				20101249	hap 40	C2		KM675156	
				20101250	hap 40	C2		KM675156	
				20101251	hap 40	C2		KM675156	
15	118.46	28.33	27	20110301	hap 56	C2		KM675172	
				20110302	hap 56	C2		KM675172	
				20110303	hap 56	C2		KM675172	
				20110304	hap 56	C2		KM675172	
				20110305	hap 56	C2		KM675172	
				20110306	hap 56	C2		KM675172	
				20110307	hap 56	C2	III	KM675172	KM675083
				20110309	hap 56	C2		KM675172	
				20110310	hap 56	C2		KM675172	
				20110311	hap 56	C2		KM675172	
				20110311	hap 56	C2		KM675172	
				20110312	hap 56	C2		KM675172	

				20110313	hap 56	C2		KM675172	
				20110314	hap 56	C2		KM675172	
				20110315	hap 56	C2		KM675172	
				20110316	hap 56	C2		KM675172	
				20110317	hap 56	C2		KM675172	
				20110318	hap 56	C2		KM675172	
				20110319	hap 56	C2		KM675172	
				20110320	hap 56	C2		KM675172	
				20110321	hap 56	C2		KM675172	
				20110322	hap 56	C2		KM675172	
				20110323	hap 56	C2		KM675172	
				20110324	hap 56	C2		KM675172	
				20110325	hap 56	C2		KM675172	
				20110326	hap 56	C2	III	KM675172	KM675083
				20110327	hap 56	C2		KM675172	
16	118	30.32	14	20101510	hap 52	C2	III	KM675168	KM675063
				20101511	hap 52	C2		KM675168	
				20101515	hap 52	C2		KM675168	
				20101520	hap 52	C2		KM675168	
				20101530	hap 52	C2		KM675168	
				20101531	hap 52	C2		KM675168	
				20101532	hap 52	C2		KM675168	
				20101533	hap 52	C2		KM675168	
				20101534	hap 52	C2		KM675168	
				20101535	hap 52	C2		KM675168	
				20101536	hap 52	C2		KM675168	

					20101507	hap 52	C2	III	KM675168	KM675089
					20101508	hap 52	C2		KM675168	
					20101509	hap 52	C2		KM675168	
17		113.76	29.51	28	20101101	hap 46	C2		KM675162	
					20101102	hap 42	C2	III	KM675158	KM675062
					20101103	hap 46	C2		KM675162	
					20101104	hap 44	C2	III	KM675160	KM675062
					20101106	hap 47	C2		KM675163	
					20101107	hap 43	C2	III	KM675159	KM675062
					20101108	hap 45	C2		KM675161	
					20101109	hap 45	C2		KM675161	
					20101428	hap 45	C2		KM675161	
					20101429	hap 45	C2		KM675161	
					20101430	hap 48	C2		KM675164	
					20101432	hap 48	C2		KM675164	
					20101433	hap 45	C2		KM675161	
					20101434	hap 45	C2		KM675161	
					20101435	hap 45	C2	III	KM675161	KM675062
					20101436	hap 45	C2		KM675161	
					20101437	hap 45	C2		KM675161	
					20101438	hap 45	C2		KM675161	
					20101439	hap 45	C2		KM675161	
					20101440	hap 45	C2		KM675161	
					20101441	hap 48	C2		KM675164	
					20101442	hap 45	C2		KM675161	
					20101443	hap 48	C2		KM675164	

					20101444	hap 48	C2		KM675164	
					20101445	hap 49	C2		KM675165	
					20101446	hap 45	C2		KM675161	
					20101447	hap 48	C2		KM675164	
					20101448	hap 48	C2		KM675164	
18		116.07	30.74	30	1004080	hap 51	C2		KM675167	
					1004081	hap 51	C2		KM675167	
					1004082	hap 51	C2	III	KM675167	KM675065
					1004083	hap 51	C2		KM675167	
					1004084	hap 51	C2		KM675167	
					1004085	hap 51	C2		KM675167	
					1004086	hap 51	C2		KM675167	
					1004087	hap 51	C2		KM675167	
					1004088	hap 51	C2		KM675167	
					1004089	hap 51	C2		KM675167	
					1004090	hap 51	C2	III	KM675167	KM675065
					1004091	hap 51	C2		KM675167	
					1004092	hap 51	C2		KM675167	
					1004093	hap 51	C2		KM675167	
					1004094	hap 51	C2		KM675167	
					1004095	hap 51	C2		KM675167	
					1004096	hap 51	C2		KM675167	
					1004097	hap 51	C2		KM675167	
					1004098	hap 51	C2		KM675167	
					1004099	hap 51	C2		KM675167	
					1004100	hap 51	C2		KM675167	

				1004101	hap 51	C2		KM675167	
				1004102	hap 51	C2		KM675167	
				1004103	hap 51	C2	III	KM675167	KM675065
				1004104	hap 51	C2		KM675167	
				1004105	hap 51	C2		KM675167	
				1004106	hap 51	C2		KM675167	
				1004107	hap 51	C2		KM675167	
				1004108	hap 51	C2		KM675167	
				1004109	hap 51	C2		KM675167	
19	115.41	31.38	26	20101276	hap 55	C2		KM675171	
				20101277	hap 55	C2	III	KM675171	KM675061
				20101278	hap 55	C2		KM675171	
				20101279	hap 55	C2	III	KM675171	KM675061
				20101280	hap 55	C2		KM675171	
				20101281	hap 55	C2		KM675171	
				20101282	hap 55	C2		KM675171	
				20101283	hap 55	C2		KM675171	
				20101284	hap 55	C2		KM675171	
				20101285	hap 55	C2		KM675171	
				20101286	hap 55	C2		KM675171	
				20101287	hap 55	C2		KM675171	
				20101288	hap 55	C2		KM675171	
				20101289	hap 55	C2	III	KM675171	KM675061
				20101290	hap 55	C2		KM675171	
				20101291	hap 55	C2		KM675171	
				20101292	hap 55	C2		KM675171	

				20101293	hap 55	C2		KM675171	
				20101294	hap 55	C2		KM675171	
				20101295	hap 55	C2		KM675171	
				20101296	hap 55	C2		KM675171	
				20101297	hap 55	C2		KM675171	
				20101298	hap 55	C2		KM675171	
				20101300	hap 55	C2		KM675171	
				20101301	hap 55	C2		KM675171	
				20101303	hap 55	C2		KM675171	
20	118.02	27.95	21	1004059	hap 50	C2		KM675166	
				1004060	hap 50	C2		KM675166	
				1004061	hap 50	C2		KM675166	
				1004062	hap 50	C2		KM675166	
				1004063	hap 50	C2		KM675166	
				1004064	hap 50	C2	III	KM675166	KM675081
				1004065	hap 50	C2		KM675166	
				1004066	hap 50	C2		KM675166	
				1004067	hap 50	C2		KM675166	
				1004068	hap 50	C2	III	KM675166	KM675081
				1004069	hap 50	C2		KM675166	
				1004070	hap 50	C2		KM675166	
				1004071	hap 50	C2		KM675166	
				1004072	hap 50	C2		KM675166	
				1004073	hap 50	C2		KM675166	
				1004074	hap 50	C2		KM675166	
				1004075	hap 50	C2		KM675166	

				1004076	hap 50	C2		KM675166	
				1004077	hap 50	C2		KM675166	
				1004078	hap 50	C2		KM675166	
				1004079	hap 50	C2	III	KM675166	KM675090
21	117.81	29.55	12	20110329	hap 57	C1	III	KM675173	KM675067
				20110330	hap 57	C1		KM675173	
				20110331	hap 57	C1	III	KM675173	KM675067
				20110332	hap 57	C1		KM675173	
				20110333	hap 57	C1		KM675173	
				20110334	hap 57	C1		KM675173	
				20110335	hap 57	C1		KM675173	
				20110336	hap 57	C1		KM675173	
				20110337	hap 57	C1	III	KM675173	KM675067
				20110338	hap 57	C1		KM675173	
				20110339	hap 57	C1		KM675173	
				20110340	hap 57	C1		KM675173	
22	122.45	40.24	30	20120802	hap 68	C1		KM675182	KM675098
				20120803	hap 110	C1		KM675224	
				20120804	hap 105	C1		KM675219	KM675101
				20120805	hap 70	C1		KM675184	KM675103
				20120806	hap 71	C1		KM675185	KM675104
				20120807	hap 74	C1		KM675188	KM675087
				20120808	hap 75	C1		KM675189	KM675097
				20120809	hap 73	C1		KM675187	KM675105
				20120810	hap 77	C1		KM675191	
				20120811	hap 69	C1		KM675183	KM675096

				20120812	hap 122	C1	KM675236	
				20120813	hap 111	C1	KM675225	
				20120814	hap 123	C1	KM675237	
				20120815	hap 110	C1	KM675224	
				20120816	hap 78	C1	KM675192	
				20120817	hap 72	C1	KM675186	KM675099
				20120818	hap 71	C1	KM675185	
				20120819	hap 104	C1	KM675218	KM675100
				20120820	hap 76	C1	KM675190	KM675076
				20120821	hap 122	C1	KM675236	
				20120822	hap 112	C1	KM675226	
				20120823	hap 106	C1	KM675220	
				20120824	hap 102	C1	KM675216	KM675076
				20120825	hap 103	C1	KM675217	KM675091
				20120826	hap 107	C1	KM675221	
				20120827	hap 114	C1	KM675228	
				20120828	hap 109	C1	KM675223	
				20120829	hap 108	C1	KM675222	
				20120830	hap 113	C1	KM675227	
				20120831	hap 79	C1	KM675193	KM675102
23	122.36	40.03	3	20120834	hap 120	C1	KM675234	KM675109
				20120835	hap 84	C1	KM675198	KM675084
				20120836	hap 120	C1	KM675234	
24	122.48	40.09	3	20120837	hap 116	C1	KM675230	KM675107
				20120838	hap 115	C1	KM675229	KM675108
				20120839	hap 102	C1	KM675216	

25		115.47	39.63	1	20120801	hap 119	C1		KM675233	KM675078
26		116.45	40.5	28	20120832	hap 81	C1		KM675195	
					20120833	hap 81	C1		KM675195	KM675078
					20120834	hap 86	C1		KM675200	
					20120835	hap 81	C1		KM675195	
					20120836	hap 81	C1		KM675195	
					20120837	hap 118	C1		KM675232	KM675079
					20120838	hap 81	C1		KM675195	
					20120839	hap 86	C1		KM675200	
					20120840	hap 80	C1		KM675194	
					20120841	hap 81	C1		KM675195	
					20120842	hap 118	C1		KM675232	
					20120843	hap 85	C1		KM675199	KM675079
					20120845	hap 87	C1		KM675201	
					20120846	hap 83	C1		KM675197	KM675078
					20120847	hap 87	C1		KM675201	
					20120848	hap 82	C1		KM675196	KM675078
					20120849	hap 87	C1		KM675201	
					20120850	hap 80	C1		KM675194	
					20120851	hap 83	C1		KM675197	
					20120852	hap 80	C1		KM675194	KM675079
					20120853	hap 80	C1		KM675194	
					20120854	hap 81	C1		KM675195	
					20120855	hap 83	C1		KM675197	
					20120856	hap 88	C1		KM675202	KM675078
					20120857	hap 117	C1		KM675231	KM675078

				20120858	hap 121	C1		KM675235	
				20120859	hap 81	C1		KM675195	
				20120860	hap 80	C1		KM675194	
27	108.74	30.19	30	20110901	hap 65	B2	II	KM675181	KM675088
				20110902	hap 65	B2	II	KM675181	KM675088
				20110903	hap 65	B2		KM675181	
				20110904	hap 65	B2		KM675181	
				20110905	hap 65	B2		KM675181	
				20110906	hap 65	B2		KM675181	
				20110907	hap 65	B2	II	KM675181	KM675088
				20110908	hap 65	B2		KM675181	
				20110909	hap 65	B2		KM675181	
				20110910	hap 65	B2		KM675181	
				20110911	hap 65	B2		KM675181	
				20110912	hap 65	B2		KM675181	
				20110913	hap 65	B2		KM675181	
				20110914	hap 65	B2		KM675181	
				20110915	hap 65	B2		KM675181	
				20110916	hap 65	B2		KM675181	
				20110917	hap 65	B2		KM675181	
				20110918	hap 65	B2		KM675181	
				20110919	hap 65	B2		KM675181	
				20110920	hap 65	B2		KM675181	
				20110921	hap 65	B2		KM675181	
				20110922	hap 65	B2		KM675181	
				20110923	hap 65	B2		KM675181	

					20110924	hap 65	B2		KM675181	
					20110925	hap 65	B2		KM675181	
					20110926	hap 65	B2		KM675181	
					20110927	hap 65	B2		KM675181	
					20110928	hap 65	B2		KM675181	
					20110929	hap 65	B2		KM675181	
					20110930	hap 65	B2		KM675181	
28		107	34.18	32	20101537	hap 62	B2	II	KM675178	KM675064
					20101538	hap 59	B2		KM675175	
					20101539	hap 59	B2		KM675175	
					20101540	hap 59	B2	III	KM675175	KM675073
					20101541	hap 59	B2	II	KM675175	KM675064
					20101542	hap 59	B2		KM675175	
					20101543	hap 59	B2		KM675175	
					20101544	hap 59	B2		KM675175	
					20101545	hap 59	B2	II	KM675175	KM675064
					20101546	hap 59	B2		KM675175	
					20101547	hap 59	B2		KM675175	
					20101548	hap 59	B2		KM675175	
					20101549	hap 59	B2		KM675175	
					20101550	hap 59	B2		KM675175	
					20101551	hap 59	B2		KM675175	
					20101552	hap 59	B2		KM675175	
					20101553	hap 59	B2		KM675175	
					20101554	hap 59	B2		KM675175	
					20101555	hap 59	B2		KM675175	

				20101556	hap 59	B2		KM675175	
				20101557	hap 59	B2		KM675175	
				20101558	hap 59	B2		KM675175	
				20101559	hap 59	B2		KM675175	
				20101560	hap 59	B2		KM675175	
				20101561	hap 59	B2		KM675175	
				20101562	hap 59	B2		KM675175	
				20101563	hap 59	B2		KM675175	
				20101564	hap 59	B2		KM675175	
				20101595	hap 59	B2		KM675175	
				20101596	hap 59	B2		KM675175	
				20101597	hap 59	B2		KM675175	
				20101598	hap 59	B2	II	KM675175	KM675064
29	107.96	33.71	22	20101479	hap 58	B2	II	KM675174	KM675064
				20101480	hap 58	B2		KM675174	
				20101481	hap 58	B2		KM675174	
				20101482	hap 58	B2		KM675174	
				20101483	hap 58	B2	II	KM675174	KM675064
				20101484	hap 58	B2		KM675174	
				20101485	hap 58	B2		KM675174	
				20101486	hap 58	B2		KM675174	
				20101487	hap 58	B2		KM675174	
				20101488	hap 58	B2		KM675174	
				20101489	hap 58	B2		KM675174	
				20101490	hap 58	B2		KM675174	
				20101491	hap 58	B2		KM675174	

				20101492	hap 58	B2		KM675174	
				20101494	hap 58	B2		KM675174	
				20101495	hap 58	B2		KM675174	
				20101496	hap 58	B2		KM675174	
				20101497	hap 58	B2		KM675174	
				20101499	hap 58	B2		KM675174	
				20101502	hap 58	B2		KM675174	
				20101503	hap 61	B2	II	KM675177	KM675064
				20101506	hap 58	B2		KM675174	
30	108	33.82	3	20101407	hap 63	B1	II	KM675179	KM675069
				20101409	hap 64	B1		KM675180	
				20101426	hap 63	B1	II	KM675179	KM675068
31	106.99	34.27	24	20101567	hap 60	B2	II	KM675176	KM675064
				20101569	hap 60	B2	II	KM675176	KM675064
				20101571	hap 60	B2	II	KM675176	KM675064
				20101572	hap 60	B2	II	KM675176	KM675064
				20101573	hap 60	B2	II	KM675176	KM675064
				20101574	hap 60	B2	II	KM675176	KM675064
				20101575	hap 60	B2		KM675176	
				20101576	hap 60	B2		KM675176	
				20101577	hap 60	B2	II	KM675176	KM675064
				20101578	hap 60	B2		KM675176	
				20101579	hap 60	B2	II	KM675176	KM675064
				20101581	hap 60	B2		KM675176	
				20101582	hap 60	B2	II	KM675176	KM675064
				20101583	hap 60	B2	II	KM675176	KM675064

				20101584	hap 60	B2	II	KM675176	KM675064
				20101585	hap 60	B2	II	KM675176	KM675064
				20101586	hap 60	B2	II	KM675176	KM675064
				20101587	hap 60	B2	II	KM675176	KM675064
				20101588	hap 60	B2	II	KM675176	KM675064
				20101588					KM675064
				20101590	hap 60	B2	II	KM675176	KM675064
				20101591	hap 60	B2	II	KM675176	KM675064
				20101592	hap 60	B2	II	KM675176	KM675064
				20101594	hap 60	B2	II	KM675176	KM675064
32	111.84	34.93	27	20120862	hap 91	B1		KM675205	
				20120863	hap 91	B1	II	KM675205	KM675064
				20120864	hap 91	B1		KM675205	
				20120865	hap 92	B1		KM675206	
				20120866	hap 91	B1		KM675205	
				20120867	hap 91	B1		KM675205	
				20120868	hap 90	B1	II	KM675204	KM675064
				20120869	hap 101	B2		KM675215	
				20120870	hap 92	B1		KM675206	
				20120871	hap 91	B1		KM675205	
				20120872	hap 92	B1		KM675206	
				20120874	hap 92	B1		KM675206	
				20120876	hap 91	B1		KM675205	
				20120878	hap 92	B1		KM675206	
				20120879	hap 101	B2	II	KM675215	KM675064
				20120880	hap 92	B1		KM675206	

				20120881	hap 100	B1	II	KM675214	KM675064
				20120882	hap 91	B1		KM675205	
				20120883	hap 92	B1	II	KM675206	KM675064
				20120884	hap 91	B1		KM675205	
				20120885	hap 124	B2	II	KM675238	KM675064
				20120886	hap 92	B1		KM675206	
				20120888	hap 92	B1		KM675206	
				20120889	hap 92	B1		KM675206	
				20120890	hap 101	B2		KM675215	
				20120922	hap 91	B1		KM675205	
				20120923	hap 92	B1		KM675206	
33	111.69	35.33	29	20120892	hap 96	B2		KM675210	
				20120893	hap 95	B2	II	KM675209	KM675092
				20120894	hap 97	B2		KM675211	
				20120895	hap 95	B2		KM675209	
				20120896	hap 97	B2		KM675211	
				20120897	hap 97	B2		KM675211	
				20120898	hap 95	B2		KM675209	
				20120899	hap 96	B2		KM675210	
				20120900	hap 97	B2		KM675211	
				20120901	hap 96	B2		KM675210	
				20120902	hap 97	B2		KM675211	
				20120903	hap 98	B2		KM675212	
				20120904	hap 91	B1		KM675205	
				20120905	hap 98	B2	II	KM675212	KM675094
				20120906	hap 97	B2	II	KM675211	KM675064

				20120907	hap 98	B2		KM675212	
				20120908	hap 96	B2	II	KM675210	KM675093
				20120909	hap 97	B2		KM675211	
				20120910	hap 97	B2		KM675211	
				20120911	hap 97	B2		KM675211	
				20120913	hap 96	B2		KM675210	
				20120914	hap 96	B2		KM675210	
				20120915	hap 96	B2		KM675210	
				20120916	hap 96	B2		KM675210	
				20120917	hap 99	B2	II	KM675213	KM675094
				20120918	hap 96	B2		KM675210	
				20120919	hap 97	B2		KM675211	
				20120920	hap 97	B2		KM675211	
				20120921	hap 97	B2		KM675211	
34	111.02	30.8	16	20120924	hap 94	B2		KM675208	KM675064
				20120925	hap 93	B2	II	KM675207	
				20120926	hap 93	B2		KM675207	
				20120927	hap 94	B2		KM675208	
				20120928	hap 93	B2		KM675207	
				20120929	hap 94	B2		KM675208	
				20120930	hap 93	B2		KM675207	KM675064
				20120931	hap 94	B2	II	KM675208	
				20120932	hap 94	B2		KM675208	
				20120933	hap 94	B2		KM675208	
				20120934	hap 93	B2		KM675207	KM675064
				20120935	hap 94	B2	II	KM675208	

					20120936	hap 94	B2		KM675208	
					20120937	hap 94	B2		KM675208	
					20120938	hap 94	B2		KM675208	
					20120939	hap 94	B2		KM675208	
35		113.92	31.86	29	20120940	hap 89	A		KM675203	
					20120941	hap 89	A		KM675203	
					20120942	hap 89	A	I	KM675203	KM675095
					20120943	hap 89	A		KM675203	
					20120944	hap 89	A		KM675203	
					20120945	hap 89	A		KM675203	
					20120946	hap 89	A		KM675203	
					20120947	hap 89	A		KM675203	
					20120948	hap 89	A		KM675203	
					20120949	hap 89	A		KM675203	
					20120950	hap 89	A		KM675203	
					20120951	hap 89	A		KM675203	
					20120952	hap 89	A		KM675203	
					20120953	hap 89	A		KM675203	
					20120954	hap 89	A	I	KM675203	KM675095
					20120955	hap 89	A		KM675203	
					20120956	hap 89	A		KM675203	
					20120957	hap 89	A		KM675203	
					20120958	hap 89	A		KM675203	
					20120959	hap 89	A		KM675203	
					20120960	hap 89	A		KM675203	
					20120961	hap 89	A		KM675203	

			20120962	hap 89	A	KM675203	
			20120963	hap 89	A	KM675203	
			20120964	hap 89	A	KM675203	
			20120965	hap 89	A	KM675203	
			20120966	hap 89	A	KM675203	
			20120967	hap 89	A	KM675203	
			20120968	hap 89	A	KM675203	
	36	2		hap 66	A	AB198971	
				hap 67	A	AB236730	
Outgroup							
R.lagowaskii		4	20101595	hap 126		KM675117	
			20101596				KM675112
			20101597	hap 127		KM675118	KM675113
			20101608	hap 128		KM675119	
