# Supplementary material for: Geological events and Pliocene climate fluctuations explain the phylogeographical pattern of the cold water fish Rhynchocypris oxycephalus (Cypriniformes: Cyprinidae) in China
Source: BMC Evol Biol. 2014 Oct 25;14:225. doi: 10.1186/s12862-014-0225-9 (PMC4219125; doi:10.1186/s12862-014-0225-9)
Supplement: Additional file 2: Figure S1. — Mismatch distribution for each R. oxycephalus matriline. The abscissa indicates the number of pairwise differences between compared sequences. The ordinate is the frequency for each value. Histograms are the observed frequencies of pairwise divergences among sequences and the line refers to the expectation under the model of population expansion. (a–d) Mismatch distributions for the matrilines B1, B2, C1, and C2, respectively. [file 12862_2014_225_MOESM2_ESM.pdf]

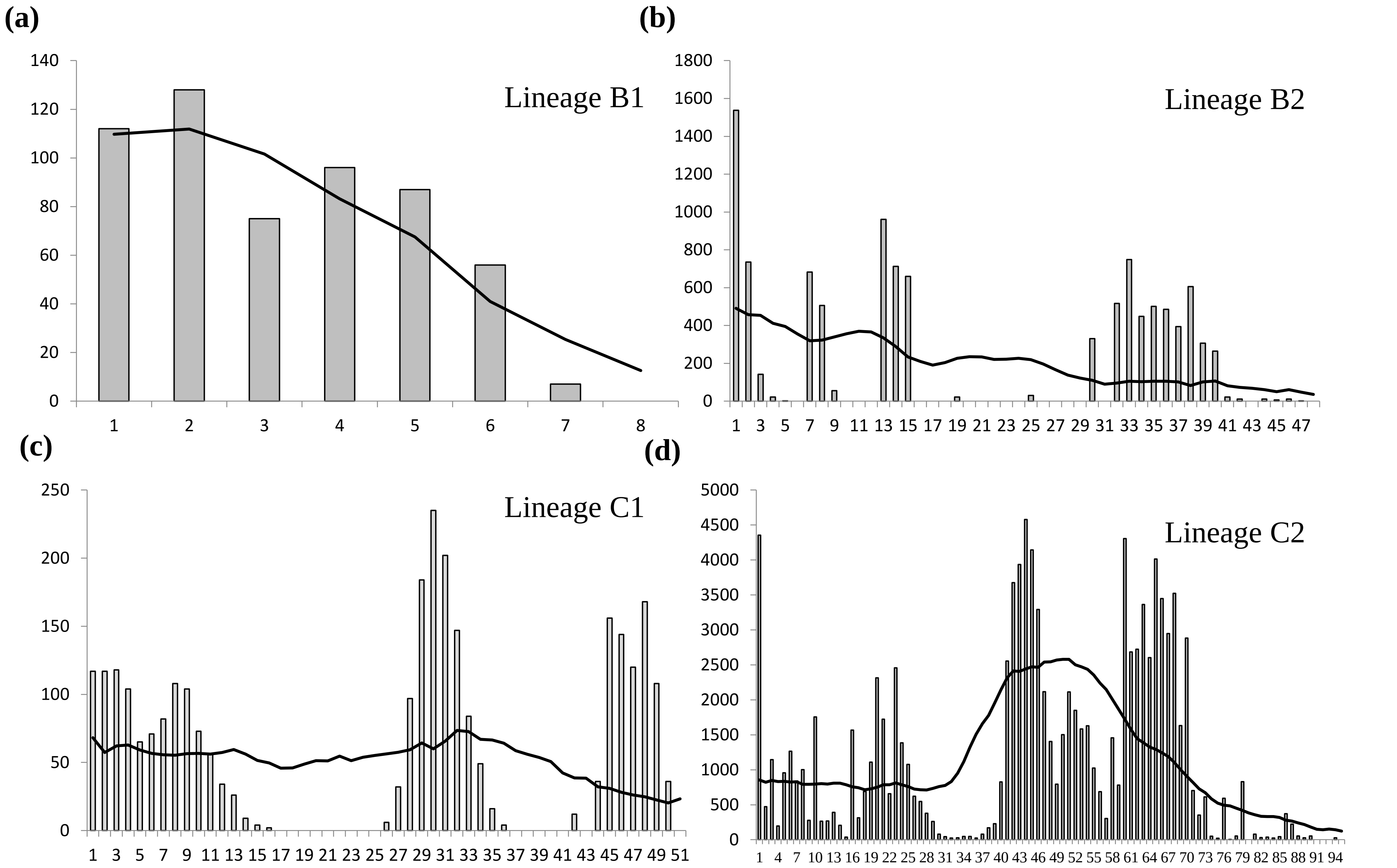

Figure S1 Mismatch distribution for each matriline of *R. oxycephalus*. The abscissa indicates the number of pairwise differences between compared sequences. The ordinate is the frequency for each value. Histograms are the observed frequencies of pairwise divergences among sequences and the line refers to the expectation under the model of population expansion. (a–d) Mismatch distribution for the matrilines B1, B2, C1 and C2, respectively.
