# Supplementary material for: Geological events and Pliocene climate fluctuations explain the phylogeographical pattern of the cold water fish Rhynchocypris oxycephalus (Cypriniformes: Cyprinidae) in China
Source: BMC Evol Biol. 2014 Oct 25;14:225. doi: 10.1186/s12862-014-0225-9 (PMC4219125; doi:10.1186/s12862-014-0225-9)
Supplement: Additional file 3: Figure S2. — Bayesian skyline plot estimated by BEAST for each R. oxycephalus matriline. X-axis, time in millions of years; y-axis, effective population size (units = Neτ, the product of effective population size and generation length in millions of years). The mean estimate and both 95% HPD limits are indicated. (a–d) Bayesian skyline plots for matrilines B1, B2, C1, and C2, respectively. [file 12862_2014_225_MOESM3_ESM.pdf]

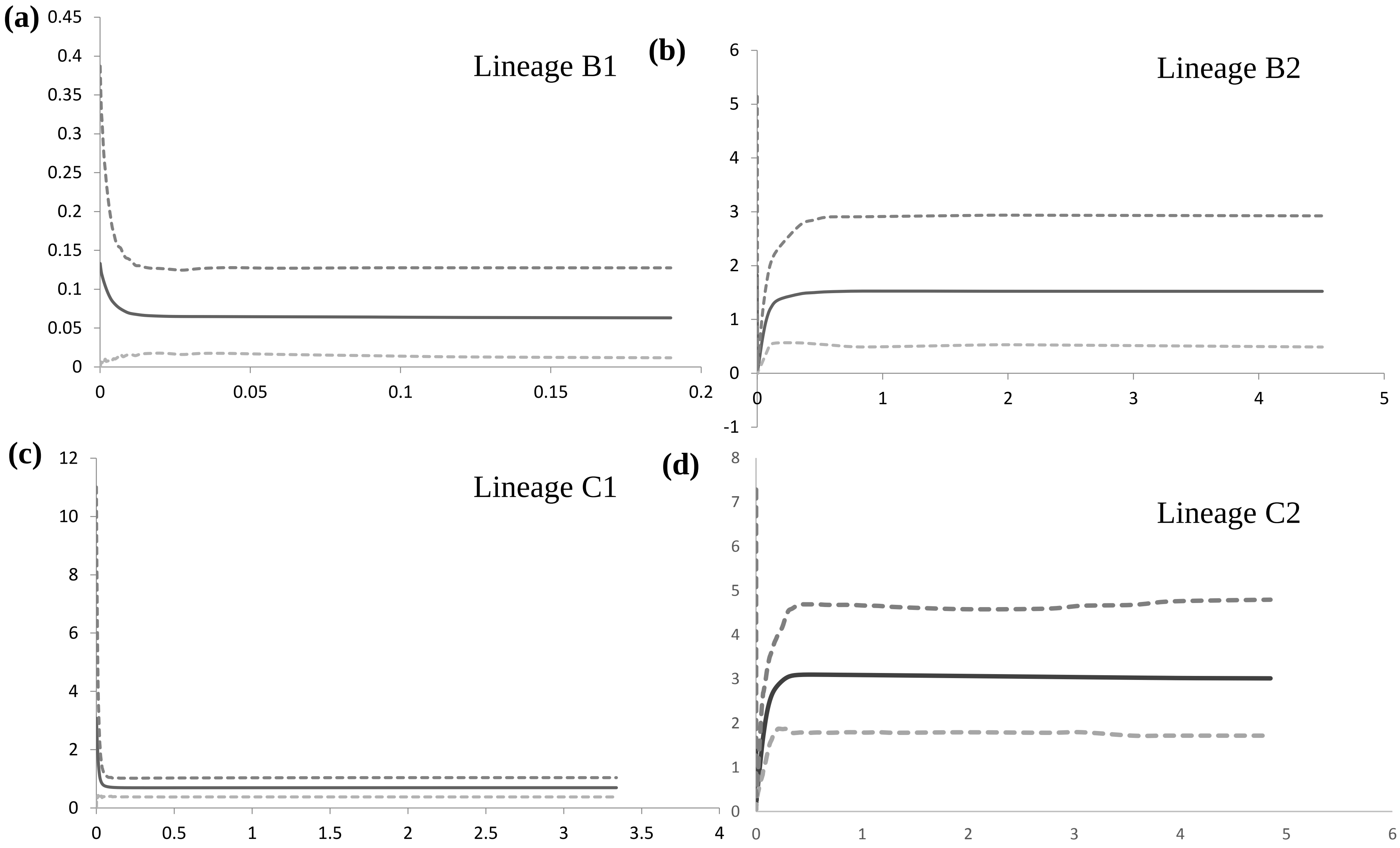

Figure S2 Bayesian skyline plot estimated by BEAST for each matriline of *R. oxycephalus*: x-axis, time in millions of years; y-axis, effective population size (units = Neτ, the product of effective population size and generation length in millions of years). The mean estimate and both 95% HPD limits are indicated. (a–d) Bayesian skyline plots for matrilines B1, B2, C1 and C2, respectively.
